# Supplementary material for: An electron transfer competent structural ensemble of membrane-bound cytochrome P450 1A1 and cytochrome P450 oxidoreductase
Source: Commun Biol. 2021 Jan 8;4:55. doi: 10.1038/s42003-020-01568-y (PMC7794467; doi:10.1038/s42003-020-01568-y)
Supplement: Supplementary file 2 — Description of Additional Supplementary Files [file 42003_2020_1568_MOESM2_ESM.pdf]

Description of Additional Supplementary Files

File name: Supplementary Data 1
Description: Excel sheet containing a list of all encounter complexes obtained in the Brownian dynamics rigid body docking simulations for the globular domains of CYP 1A1 and CPR (referred to in 'Results and Discussion, Electrostatic steering of the CYP 1A1 and the CPR FMN domains leads to encounter complex formation').

File name: Supplementary Data 2
Description: Source data underlying plots shown in figures 3, 4, and 5i and Table 1.
